# Draft Genome Sequence of Plesiomonas shigelloides Strain zfcc0051 (Phylum *Proteobacteria*)

**DOI:** 10.1128/mra.00074-22

**Published:** 2022-05-31

**Authors:** Zoe K. VanderHoek, Addison M. Browning, Quinn Washburn, Micheal L. Kent, Thomas J. Sharpton, Christopher A. Gaulke

**Affiliations:** a Department of Microbiology, Oregon State University, Corvallis, Oregon, USA; b Department of Statistics, Oregon State University, Corvallis, Oregon, USA; c Department of Pathobiology, University of Illinois Urbana-Champaign, Urbana, Illinois, USA; d Carl R. Woese Institute for Genomic Biology, University of Illinois Urbana-Champaign, Urbana, Illinois, USA; SIPBS, University of Strathclyde

## Abstract

Here, we report a draft genome sequence of Plesiomonas shigelloides strain zfcc0051, an isolate derived from zebrafish (Danio rerio) feces. The genome consists of 115 contigs (>500 bp) and has a total assembly length of 4,041,537 bases.

## ANNOUNCEMENT

Plesiomonas shigelloides is a facultative, anaerobic, non-spore-forming, Gram-negative bacillus frequently found in freshwater ecosystems, freshwater fish guts, and occasionally, aquatic mammals (1, 2). In laboratory and wild zebrafish, Plesiomonas shigelloides abundance is high (3), and it has been identified as a potential indicator of health (4, 5). In contrast, other reports indicate that P. shigelloides can act as a pathogen of zebrafish (2, 6); however, direct exposure with P. shigelloides does not consistently yield infection (7). One possible explanation for the conflicting evidence surrounding the associations between P. shigelloides and health is that genetic variation among isolates gives rise to strains that can differentially impact fish physiology. However, the limited number of publicly available genomes for *Plesiomonas* spp. complicates comparative genomic investigations that could shed light on this question. To facilitate such investigations, we sequenced and annotated a microbial isolate from zebrafish gut associated with Plesiomonas shigelloides.

A fresh fecal sample was collected from a 9-L tank containing approximately 30 12-month-old tropical 5D line laboratory-raised zebrafish (Corvallis, Oregon), aseptically syringe-homogenized, serially diluted, plated on brain heart infusion (BHI) agar (BD), and incubated at 27°C. Individual colonies were picked and further isolated with two rounds of streak plating. One isolated colony was used to inoculate BHI broth and was incubated at 27°C for 24 h. Bacterial DNA was isolated from this pure culture using the UltraClean microbial DNA isolation kit (Qiagen) and subsequently used for 16S amplicon and genome sequencing. A 1,400-bp fragment of the 16S rRNA gene was amplified using 27F and 1492R (8) primers, purified with the UltraClean PCR cleanup kit (Qiagen), sequenced on an ABI 3730 DNA analyzer (Applied Biosystems), and aligned to the NCBI 16S rRNA database using BLAST v2.9.0 (9). The best alignment (97.4% identity) for this culture was associated with Plesiomonas shigelloides (GenBank version number NR_117763.1). Next, we generated and sequenced a bacterial DNA library using the Nextera XT kit and a MiSeq instrument. The resulting 3,234,209 300-bp paired-end reads were subsequently filtered and trimmed using ea-utils v1.04 (10). SPAdes v3.1.1 (11) was used to assemble a 4,041,537-bp genome in 604 contigs (115 to contigs >500 bp) with an *N*_50_ value of 187,457 (all contigs), 51.7% GC content, and 480× coverage. Genome completeness was assessed as 100% by examining the presence of 40 universal marker genes (CheckM v1.1.2) (12). Similar to other P. shigelloides strains, Prodigal v2.6.3 (13) and the NCBI Prokaryotic Genome Annotation Pipeline (v5.1) identified 3,952 and 3,693 protein-coding genes in our assembly, respectively. Given the identity between the 16S rRNA gene of our isolate and P. shigelloides, we used fastANI v1.32 (14) to calculate the average nucleotide identity (ANI) of our isolate and a reference P. shigelloides strain, NCTC10360 (GenBank version number GCA_900087055.1). These genomes shared 97.3% ANI, consistent with the determination that our isolate represents a novel strain of P. shigelloides.

### Data availability.

This whole-genome shotgun project has been deposited at DDBJ/ENA/GenBank under the accession number JAFNAA000000000. The version described in this paper is version JAFNAA010000000. The raw sequence reads are deposited at the Sequence Read Archive under number SRP357730. Workflow with parameters is available at https://github.com/chrisgaulke/zfcc.
